# Crossed Cerebellar Diaschisis: Three Case Reports Imaging Using a Tri-Modality PET/CT–MR System

**DOI:** 10.1097/MD.0000000000002526

**Published:** 2016-01-15

**Authors:** Shuguang Han, Xiaopeng Wang, Kai Xu, Chunfeng Hu

**Affiliations:** From the Department of Radiology and the Department of Neurology (SH, KX, CH), Affiliated Hospital of Xuzhou Medical College, Xuzhou, Jiangsu Province, PR China (XW).

## Abstract

Crossed cerebellar diaschisis (CCD) describes a depression of oxidative metabolism glucose and blood flow in the cerebellum secondary to a supratentorial lesion in the contralateral cerebral hemisphere. PET/MR has the potential to become a powerful tool for demonstrating and imaging intracranial lesions .We herein report 3 cases of CCD imaging using a tri-modality PET/CT–MR set-up for investigating the value of adding MRI rather than CT to PET in clinical routine.

We describe 3 patients with CCD and neurological symptoms in conjunction with abnormal cerebral fluorodeoxyglucose (FDG) positron emission tomography/computed tomography-magnetic resonance imaging (PET/CT–MR) manifestations including arterial spin-labeling (ASL) and T2-weighted images. In all, ^18^FDG-PET/CT detected positive FDG uptake in supratentorial lesions, and hypometabolism with atrophy in the contralateral cerebellum. More than that, hybrid PET/MRI provided a more accurate anatomic localization and ASL indicated disruption of the cortico-ponto-cerebellar pathway.

Using pathology or long-term clinical follow-up to confirm the PET and ASL findings, the supratentorial lesions of the 3 patients were respectively diagnosed with cerebral infarction, recurrent glioma, and metastasis.

The reports emphasize the signiﬁcance of multimodality radiological examinations. Multimodality imaging contributes to proper diagnosis, management, and follow-up of supratentorial lesions with CCD.

## INTRODUCTION

Crossed cerebellar diaschisis (CCD) is characterized by loss of functional activity and metabolism in the cerebellum contralateral to the supratentorial lesion. This phenomenon has been observed in patients with cerebral infarction,^1^ supratentorial tumors,^2^ epilepsy,^3^ encephalitis,^4,5^ and as a result of disruption of the cortico-ponto-cerebellar pathway. It has been previously described on single-photon emission CT (SPECT) and positron emission tomography (PET) scans but has rarely been reported on PET/ magnetic resonance imaging (MRI) examinations.

The trimodality PET/CT–MR system offers a novel technique using the 3 different imaging modalities in 1 patient virtually at the same time.^6^ In this report, we aimed to characterize CCD with different supratentorial lesions by PET/MRI in comparison with PET/CT. This study was approved by the Institutional Review Board.

## CASE REPORTS

### Case 1

A 61-year-old woman was admitted to our hospital for unilateral limb weakness after suffering a stroke. She had a 15-year history of hypertension and type 2 diabetes mellitus controlled by subcutaneous insulin injection. The neurologic examination revealed that the left nasolabial groove became shallow and the tongue was deviated to right, as well as both Babinski sign positive, without sign of meningeal irritation.

**FIGURE 1:**
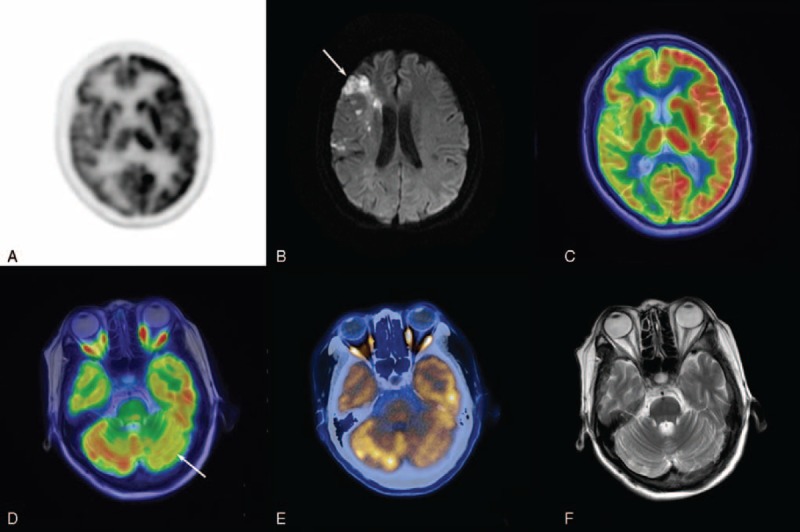
61-year-old woman after suffering a stroke. (A) Axial PET images revealed a marked reduction in cortical and subcortical glucose metabolism in the right hemisphere. (B) On axial DWI, the frontal lesion demonstrated high signal illustrating a significant diffusion restriction (arrow). (C) Axial FDG PET fused with FSE T2WI of this patient showed the right hemispheric infarction with hypometabolism. (D) Fused PET/MRI images demonstrated hypometabolism in the contralteral cerebellar hemisphere (arrow). (E) Fused PET/CT images also demonstrated hypometabolism in the contralteral cerebellar hemisphere but with low soft-tissue resolution. (F) Axial FSE T2WI confirmed no significant changes or sign of atrophy in the cerebellum. CT = computed tomography, DWI = diffusion-weighted imaging, FDG = fluorodeoxyglucose, FSE = fast spin echo, MRI = magnetic resonance imaging, PET = positron emission tomography.

Axial PET images revealed a marked reduction in cortical and subcortical glucose metabolism in the right hemisphere (Figure 1A). On axial diffusion-weighted image, the frontal lesion demonstrated high signal illustrating a significant diffusion restriction (Figure 1B). This evaluation was performed on a hybrid PET/CT-MRI scanner combining a 3.0 T MRI (Discovery 750w 3T, GE Healthcare, Waukesha, USA) and a TOF PET/CT (Time of flight, Discovery 690, GE Healthcare, Waukesha, USA). PET/CT, PET/MRI, PET-only or CT-only images can be acquired on a dedicated review workstation (Advantage Windows 4.6, GE Healthcare). Axial FDG PET fused with FSE T2 images of this patient showed the infarction of right hemispheric (Figure 1C). Fused PET/MRI (Figure 1D) and PET/CT (Figure 1E) images demonstrated hypometabolism in the contralteral cerebellar hemisphere. Both modalities exhibited similar diagnostic image quality whereas the former offered higher soft-tissue contrast. The axial FSE T2 image confirmed no significant changes or sign of atrophy in the cerebellum (Figure 1F). The patient was eventually proved to acute cerebral infarction associated with CCD.

### Case 2

A 49-year-old man, after a resection of frontal lobe glioma 2 years ago, was admitted to our hospital with an alalia and secondary epilepsy. The symptoms gradually worsened, and epileptic seizures increasingly serious, the last an average of once every 2 months to attack. The neurologic examination showed inarticulate speech and deviation of mouth angle, with right Babinski sign positive.

**FIGURE 2:**
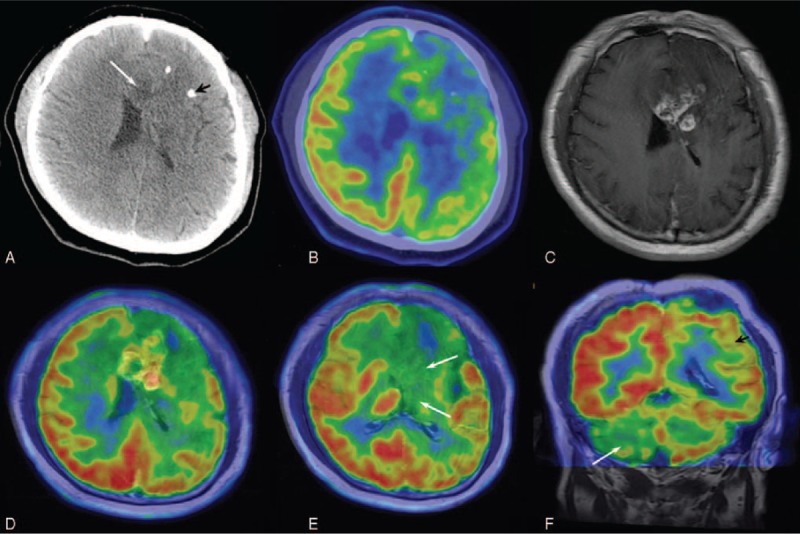
A 49-year-old man confirmed recurrence after a resection of frontal lobe glioma. (A) Axial CT images revealed ill-defined hypodensity in the surgical site (arrow), and that scattered calcified speckles around the lesion (arrowhead). (B) Postoperative site showed predominantly reduced uptake as seen on the axial PET/CT image, with a small focus of mildly increased uptake within. (C) T1-weighted CE-MRI exhibited in the bilateral frontal lobe an area of contrast enhancing lesion suggestive for recurrence of glioma. (D) Fused PET/MRI images demonstrated focal hypermetabolism in the frontal lobe which corresponds to lesion after enhancement of gadolinium. (E) Axial FDG PET fused with T1-weighted CE-MRI of this patient also showed hypometabolism in the homolateral basal nuclei and thalamus (arrow). (F) Coronal PET/MRI images show a marked reduction in cortical and subcortical glucose metabolism in the right cerebellar hemisphere (arrowhead) associated with a hypometabolism of the contralateral hemisphere (arrow). CT = computed tomography, FDG = fluorodeoxyglucose, MRI = magnetic resonance imaging, PET = positron emission tomography.

Axial CT images revealed ill-defined hypodensity in the surgical site, and that scattered calcified speckles around the lesion (Figure 2A). Postoperative site showed predominantly reduced uptake as seen on the axial ^18^F-FDG fused PET/CT image, with a small focus of mildly increased uptake within (Figure 2B). Actually, ^18^F-FDG PET/CT was not sufficient to identify the tumor recurrence. T1-weighted CE-MRI of the brain (Figure 2C), subsequently performed with hybrid PET/CT-MRI scanner, exhibited in the bilateral frontal lobe an area of contrast enhancing lesion suggestive for recurrence of glioma, which corresponds to lesion uptake of radiotracer in the fused PET/MRI image (Figure 2D). Axial FDG PET fused with T1-weighted CE-MRI of this patient also showed hypometabolism in the homolateral basal nuclei and thalamus (Figure 2E), whereas the FSE T2 image and FLAIR MRI done at the same time demonstrated no significant change (not shown). Coronal PET/MRI images show a marked reduction in cortical and subcortical glucose metabolism in the right cerebellar hemisphere associated with a hypometabolism of the contralateral hemisphere (Figure 2F), which also confirmed a mild degree of cerebella atrophy, as slight widening of the right cerebellar hemisphere sulci. The recurrent glioma was confirmed on postoperative histopathology.

### Case 3

A 73-year-old woman was admitted to our hospital complaining of progressive dizziness and headache, slow response, lisp, vague, and right hemiplegia. An adenocarcinoma of the lung was diagnosed in October 2012 and she had undergone pulmonary lobectomy and adjuvant chemotherapy. Fifteen months later (January 2014), a follow-up brain CT scan and subsequent PET/CT-MRI revealed a solitary lesion in the left frontal lobe, highly suggestive for a brain metastasis.

**FIGURE 3:**
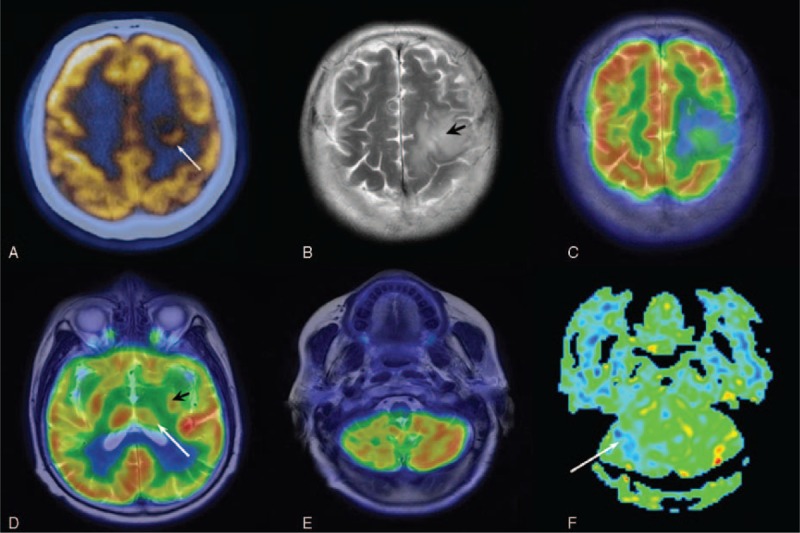
A 73-year-old woman confirmed the diagnosis of brain metastases. (A) Axial fused PET/CT showed a ring-like area of 18F-FDG uptake in the left frontal lobe with ill-defined margin (arrow). (B) Axial FSE T2WI exhibited in the left frontal lobe an area of high signal suggestive for perilesional edema (arrowhead). (C) Axial FDG PET fused with FSE T2WI showed the corresponding peritumoral edema with hypometabolism. (D) The axial FDG PET/MRI image of this patient showed hypometabolism in the homolateral basal nuclei (arrowhead) and thalamus (arrow). (E) Fused PET/MRI images show a marked reduction in cortical and subcortical glucose metabolism in the right cerebellar hemisphere. (F) The ASL perfusion image done at the same time also showed the right cerebellar hypoperfusion (arrow), in keeping with the phenomenon of CCD. ASL = arterial spin-labeling, CCD = crossed cerebellar diaschisis, FDG = fluorodeoxyglucose, MRI = magnetic resonance imaging, PET = positron emission tomography.

Transaxial fused PET/CT showed a ring-like area of ^18^F-FDG uptake in the left frontal lobe with ill-defined margin, and glucose metabolism in the bilateral cerebral cortex appear to be similar (Figure 3A). The axial FSE T2 image (Figure 3B), performed with hybrid PET/CT-MRI scanner, exhibited in the left frontal lobe an area of high signal suggestive for perilesional edema, which corresponds to low uptake of radiotracer in the fused PET/MRI image (Figure 3C). Axial FDG PET fused with T2-weighted image of this patient also showed hypometabolism in the homolateral basal nuclei and thalamus (Figure 3D), whereas the T2-weighted image alone demonstrated no specific tumor infiltration and atrophy of cortex (not shown). Fused PET/MRI images show a marked reduction in cortical and subcortical glucose metabolism in the right cerebellar hemisphere (Figure 3E). In particular, the ASL (arterial spin-labeling) perfusion image done at the same time also showed the right cerebellar hypoperfusion, in keeping with the phenomenon of crossed cerebellar diaschisis (Figure 3F). The metastasis originating from adenocarcinoma of the lung was confirmed on postoperative histopathology.

## DISCUSSION

CCD, first introduced by Monakow in 1914,^7^ is characterized by hypometabolism and functional deactivation in the cerebellar hemisphere contralateral to the supratentorial lesion. Several nuclear medicine-based studies have proven that the severity of CCD appears to be an valuable prognostic marker for recovery and treatment response.^8,9^ This phenomenon of diaschisis often resolves with time whereas the mechanisms are still unknown.

To date, most cases of CCD were reported using SPECT, PET, computed tomography perfusion,^10^ dynamic susceptibility contrast perfusion MRI ^11^ and arterial spin-labeling (ASL) perfusion technique.^12^ However, to the best of our knowledge, there has been only 1 case report that described the findings of CCD after stroke with hybrid PET-MRI imaging^13^ until now. For our cases, we initially used an advanced trimodality PET/CT–MRI system, which transferred the patient on a described dedicated shuttle from 1 room into the other.^6,14^ Advantages of this set-up include the true CT-based attenuation correction, suitable MR sequences for diagnostic purposes, and reliable PET-quantification. Molecular imaging, traditionally by PET/CT and SPECT images, demonstrate a reduced metabolism and perfusion in the cerebellum contralateral to the cortical lesions.

Exploring the relationship between lesion location, volume, and the occurrence of CCD was not the major purpose of our reports owing to the small sample. All cases emphasize the diagnosis value of functional PET/MRI images, especially in case 2, which make it easier to correlate between PET and MR images and provided a more accurate anatomic localization of the lesions than the unenhanced PET/CT images. Several publications also investigated the superiority of PET/MRI versus PET/CT in the detection and conspicuity of intracranial lesions.^15,16^ It is worth mentioning that PET images of all presented showed hypometabolism in the homolateral basal nuclei and thalamus, whereas the FSE T2 image and FLAIR MRI done at the same time demonstrated no significant change. In addition, case 3 indicates that the frontal cortical edema can also lead to CCD although the metastasis itself is small and located in the subcortical region. None of the studies reported before has, however, mentioned this phenomenon observed in CCD. Morphologic changes, in particular, crossed cerebellar atrophy (CCA), occurs only in the case 2. Tien et al^17^ indicated that CCA usually appears in conjunction with large supratentonal diseases for which many cortico-pontine-cerebellar tract fibers were interrupted.

Even more important, the PET images can be applied in conjunction with structural, perfusion-weighted, and enhanced magnetic resonance imaging by using the trimodality system. The noncontrast-enhanced perfusion sequences (ASL) acquired in case 3 well demonstrated crossed cerebellar hypoperfusion. In CCD, this hybrid technology in a single imaging session indicates the morphologic changes of the neural pathway as well as the associated metabolic and perfusion depression of the cerebellum.

In summary, we report 3 cases of CCD with different supratentorial lesions detected by a noval PET/CT–MR system. With advantages in the simultaneous assessment of the anatomic and metabolic alterations, this trimodality system seems to be an ideal tool for the detection and follow-up of CCD.
